# Influence of Solvent Polarity on Crocin Content and Surface Properties of Saffron (*Crocus sativus* L.) Extracts

**DOI:** 10.3390/molecules29215144

**Published:** 2024-10-31

**Authors:** Rachele Rocchi, Carla Di Mattia, Gaia Gabriele, Lilia Neri, Paola Pittia

**Affiliations:** 1 Istituto Zooprofilattico Sperimentale dell’Abruzzo e del Molise “G. Caporale”, Via Campo Boario, 64100 Teramo, Italy; 2 Department of Bioscience and Technology for Food, Agriculture and Environment, University of Teramo, Via Balzarini 1, 64100 Teramo, Italy; gaiagabriele@gmail.com (G.G.); lneri@unite.it (L.N.); ppittia@unite.it (P.P.)

**Keywords:** saffron, air–water surface tension, extract polarity, crocins, technological functionality

## Abstract

The saffron composition is being widely studied for authenticity and traceability, but very few works have been carried out to investigate the relationship between the chemical and physico-chemical properties of saffron solutes and their technological functionality in colloidal systems. This study aims at evaluating the surface properties of saffron extracts obtained using solvents of different polarities to achieve extracts with different compositions in terms of the pattern and content of polar and medium polarity crocins. The air–water surface was evaluated alone and in the presence of Tween 20 at different surfactant–extract ratios. Saffron extracts were able to decrease the surface tension of the aqueous phase, indicating the presence of surface-active compounds. In the mixed saffron extract–Tween 20 systems, competitive adsorption at the air–water interface occurred when the surfactant was present at a low concentration, while at concentrations higher than the CMC, Tween 20 hindered the adsorption of the extract surface-active compounds. The results highlight the interesting technological functionality of saffron extracts for applications in colloidal systems. To better exploit their use in the design and development of formulated foods, nutraceutics and pharma products, further studies are needed to unravel the relationship between the composition of saffron extracts and corresponding surface activity.

## 1. Introduction

Saffron is the dried red stigma of the *Crocus sativus* L. flower and the word saffron probably is derived from the Iranian-Arabic name of the plant, za`faran, which means yellow. Nowadays, it is cultivated largely in Europe, Turkey, Central Asia, India, and China. Saffron has been used as dyestuff, as medicament, and as a perfume; however, these applications have declined in modern times. The main market now is as a food spice, by far the world’s most expensive [1]. Saffron, in addition, has been used traditionally to treat many diseases, such as multiple cancers, neurological defects, cognitive problems, inflammatory and autoimmune diseases, as well as neural and cardiovascular inflammation [2,3,4,5,6,7,8].

The environment, climate conditions, and geographical area of cultivation, along with post-harvest drying process, which varies country by country, can affect the saffron chemical composition [9,10,11] that is characterized by a large number of nutritive and bioactive compounds, such as monoterpenoids, flavonoids, carotenoids, proteins, vitamins (riboflavin and thiamine), carbohydrates, amino acids, mineral matter, gums, and many other chemical compounds [12,13].

In the literature, a number of studies dealing with the chemical, nutritional composition, and quality classification of saffron have been carried out mainly for authenticity and traceability [14,15,16,17,18,19,20,21,22,23,24,25,26,27,28,29].

Among the key secondary metabolites of saffron, crocins, a class of carotenoids present in saffron, have attracted high interest for their main health beneficial effects as potent antioxidants that may protect cells from oxidative stress [30,31,32] Normally, carotenoids are liposoluble, but crocins are hydrosoluble carotenoids due to the presence of carbohydrate residues, such as glucose, gentiobiose, or triglucose [33]. These glycosyl esters can exist in cis- and trans- isomeric forms; some studies have demonstrated that the pattern of crocins depends on the variety, growing conditions, drying conditions, and storage [9,10,23].

Due to their structure, crocins can be considered as amphiphilic compounds that are able to exert some interfacial properties and affect the formation and stabilization of emulsified systems; however, to the best of the authors’ knowledge, very few works on crocins surface properties are available in the literature. Naess et al. (2006) [34] studied the adsorption behaviour of trans-crocin 4 in water and observed a decrease in the surface tension up to a value of 52 mN/m at the point of discontinuity for the critical micelle concentration of 0.8 mg/mL; this result was related to the ability of these molecules to migrate at the air–water interface from the bulk of the solution [30]. Moreover, crocins were proven to interact with proteins and affect significantly the surface activity of crocins and bovine serum albumin mixed systems by the formation of a complex that was more surface active than the pure compounds by themselves [35].

The interest towards the surface or interfacial activity of secondary metabolites of plant extracts (e.g., olive leaves and tea) is growing due to their potential effects on the quality and stability of colloidal systems [36,37,38,39,40,41]. The interface between two phases controls the interaction between colloidal particles. This interaction determines, in turn, the colloidal stability that is the result of the balance of different type of forces (electrostatic, Van der Waals, steric, etc.) and prevents the general breakdown in the bulk structure [42]. In food emulsions, the attainment of an adequate structure and the achievement of oxidative and physical stability upon storage represents a sensitive goal. In fact, in contrast to the behaviour observed in simple model systems, in the real emulsified ones (e.g., mayonnaise, ice cream, milk, and soft drinks), other molecules that are naturally present as ingredients or intentionally added with surface activity properties could be present and make more complicated the surface functionality of the emulsifying agents by competition for dominance at the interface [36]. These phenomena, when they occur, can either hinder or enhance the colloidal properties and stability, depending on the system [42].

Moreover, plant extracts are in general very complex systems made of components of different natures, molecular sizes and polarities [43], whose composition depends also on the extraction method applied, and in the particular case of liquid–solid extracts, the nature of the solvent can preferentially determine a different composition of the extract. This may further increase the difficulty of understanding the effects of extracts containing surface-active compounds when added as external component in colloidal systems. Currently, saffron extracts have been applied as colouring and flavouring agents, as well as for their bioactive antioxidant properties, and added in real-like and real products like dairy products, pasta, as well as beverages, etc. [44,45,46,47,48,49,50].

Esfanjani et al. (2015) [51] reported the nano-encapsulation of saffron extracts using a spray-drying technique to preserve the bioactive compounds of saffron; the W/O/W multiple emulsions were stabilized by whey protein concentrate (WPC)/polysaccharide (pectin) with a high encapsulation efficiency (EE above 93% for picrocrocin, safranal, and crocins). Najafi et al. (2021) [52] also reported the encapsulation of saffron extract in nanoliposomes of rapeseed lecithin through sonication. The encapsulation caused better protection of saffron extract components during release compared to the free saffron extract, which immediately degraded under the same conditions in PBS solution [52]. None of them, however, deepened the understanding of the relationship between chemical and physico-chemical properties of saffron extracts and their technological functionalities in food matrices as well as in model colloidal systems.

The aim of this study was, thus, to evaluate the surface properties of saffron extracts of three different products (two from Italy and one commercial blend). For each saffron sample, two different solvents were used to obtain extracts characterised by different polarities in water (H_2_O) and methanol/acetonitrile (MeOH/MeCN). Saffron samples were classified according to the ISO 3632-1:2011 procedure [53], while the extracts were characterised in terms of the crocin content and pattern. Extracts of different polarity were studied for their surface properties at the air–water interfacial layer, alone and in combination with Tween 20 at different surfactant–extract ratios to investigate any potential competitive or synergistic phenomena.

## 2. Results and Discussion

### 2.1. Quality Evaluation of Saffron

The quality of saffron is mainly related to the contents of three main bioactive compounds, namely, picrocrocin, safranal, and crocins, which are known to affect its sensory properties, such as the flavour, aroma, and colouring strength. Several factors, including climatic conditions, harvesting, the drying process, and storage, affect presence and concentration of these compounds that, thus, may vary largely in the spice [9]. In particular, the drying process directly influences enzymatic reactions of degradation/formation of important compounds, and are responsible for quality and storage of saffron [9,10,23].

Saffron has to comply with the requirements of the International Organization for Standardization (ISO 3632-1, 2011) [53] by which its quality and authenticity are commonly evaluated via parameters determined by spectrophotometric analysis.

Table 1 reports the values of the quality parameters of the three samples and the corresponding ISO category. All samples belong to the first category (i.e., higher quality), while significantly different values (*p* < 0.05) of the three quality parameters were found. In particular, the ABR2 saffron showed lower colouring, aroma, and flavour strength values than the other two products that may be related to lower contents of the corresponding three key compounds (crocins, safranal, and picrocrocin).

### 2.2. Chemical Characterization of Saffron Extracts

H_2_O (WE) and a mix of MeOH/MeCN (MPE) were used to obtain extracts with different compositions in terms of the patterns and contents of polar and medium polarity compounds. Both the WE and MPE saffron extracts were then characterized using both spectrophotometric and HPLC-DAD analyses.

#### 2.2.1. UV-Vis Analysis

A preliminary characterisation of the differently obtained saffron extracts was carried out by a UV-Vis spectrophotometry analysis at fixed wavelengths corresponding to the absorbance of the three main secondary metabolites of saffron (i.e., picrocrocin: 257 nm, safranal: 330 nm, and crocins: 440 nm), and the results are reported in Table 2. It must be pointed out that these results, despite being obtained from the water extracts, do not have a similar trend in the absorbance values as those reported in Table 1, as the latter was determined according to the ISO method, which is the result of a more complex computation that takes into account other parameters.

Regarding crocins, COMM and ABR2 MPE presented a higher (*p* < 0.05) colouring strength value than the corresponding WE, while no differences were found for the ABR1 extract. Orfanou et al. (1996) [54], who studied the differences in the colouring strengths of saffron samples extracted using different solvents, reported that the use of MeOH allows a better extraction of the colouring components (i.e., crocins and other carotenoids).

When using the same extraction solvent, no significant differences in both safranal and picrocrocin contents among samples were observed; the same result could be found when WE and MPE of the same saffron were compared. The picrocrocin recovery is extremely limited, as also reported by other authors who developed and validated a SPE procedure to obtain a better extraction of this secondary metabolite of saffron [16].

#### 2.2.2. HPLC-DAD Analysis

The HPLC-DAD analysis was carried out in order to characterise the crocin pattern and obtain a semi-quantitative content and distribution of the isomers. Table 3 reports the relative concentration of each isomer as a percentage of the total area of the chromatographic area along with the total area.

Due to the limited standard availability, only the concentration of the trans4 crocin isomer has been determined and reported in Table 3.

No significant differences were noticed between the different saffron extracts in the total crocin content, except for the COMM WE extract that showed a lower (*p* < 0.05) total crocin content. These data seem to be partly in agreement with those obtained by the spectrophotometric analysis (Table 3). A poor correlation (R^2^ = 0.51) was found between the total crocin area and absorbance at 440 nm and this could be due to the a-specific responses of the UV/Vis technique, so that every substance that absorbs at the same wavelength could contribute to the absorbance detected by the instrument.

For all the extracts under investigation, seven crocin isomers were separated and identified, and trans4 was the most abundant one, followed by trans3. The two saffron samples from Abruzzo (ABR1 and ABR2) presented a high concentration of trans4, but no significative differences were observed between the concentrations found in WE and MPE extracts.

The COMM saffron sample showed a lower concentration of trans4 than the other extracts, but a higher relative content of trans2 and trans1 for both types of extracts. This different crocin isomer pattern may reflect the effects of various processing and storage factors. Moreover, some studies highlighted that it could be related to drying that, when carried out at high temperature, promotes the loss of glucose moieties, causing a decrease in the trans4 isomer in favour of the trans3 and trans2 moieties [23].

### 2.3. Adsorption Behavior at the A/W Interface

The adsorption kinetics of the WE and MPE extracts at the air–water (*a*/*w*) interface were determined by recording the change in the surface tension (ST) value as a function of time for up to 60 min. The adsorption time was selected after preliminary evaluations carried out in a longer span time (up to 12 h) to detect the reaching of equilibrium or pseudo-equilibrium conditions and highlight any possible change in the surface tension value that may be caused by processes and/or reactions of the surfactant agents that can take place at the interface (e.g., oxidation) [55].

The use of solvents with different polarities can cause a selective extraction of compounds that can differ in their chemical and physicochemical properties, including their polarity, and this could also have an effect on the extraction of the more amphiphilic ones. Thus, a different behaviour of the molecules at the air–water and oil–water interface was expected.

**Figure 1:**
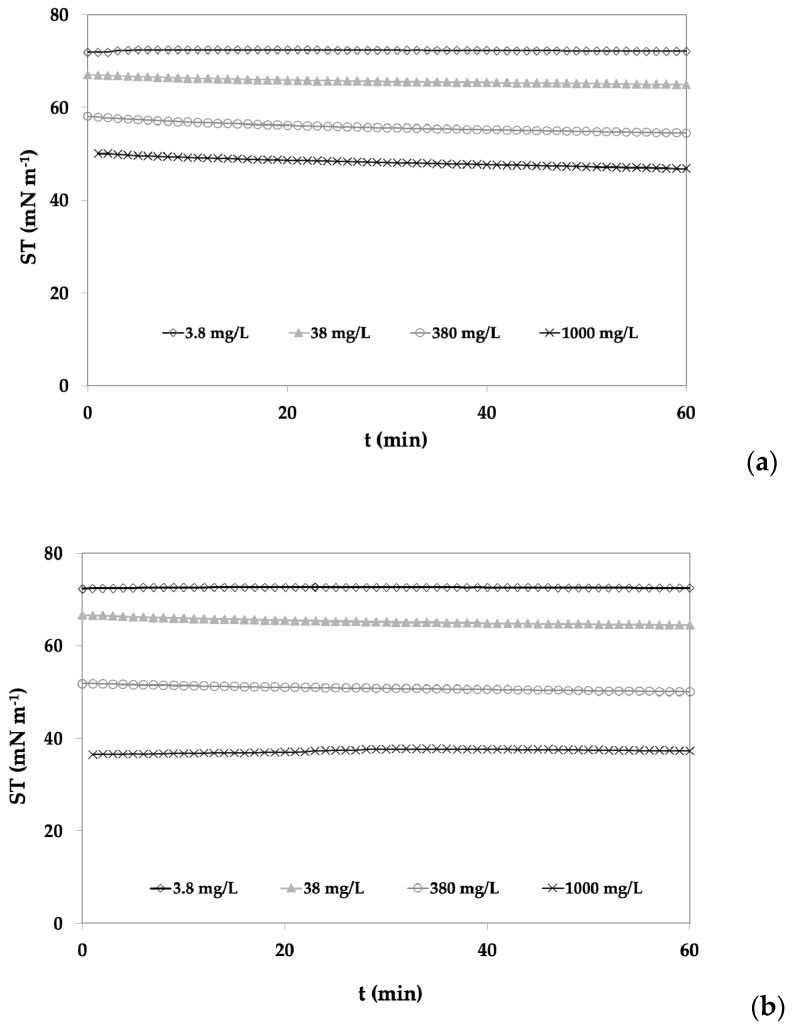
Adsorption kinetics of the saffron (**a**) WE and (**b**) MPE of the ABR1 sample at increasing concentrations.

The ST of both WE and MPE saffron extracts was determined at the *a*/*w* interface for increasing concentrations of the water phase, and in Figure 1, the adsorption kinetic of the ABR1 WE (Figure 1a) and MPE (Figure 1b) extracts are reported. Similar trends were obtained for the ABR2 and COMM samples, that on the contrary, differ in the ST values. From Figure 1a, it could be noticed that the adsorption kinetics of the surface-active components at different concentrations reached the *a*/*w* interface very quickly, as at time = 0, the surface tension values were significantly lower than water for all extract concentrations, with the exception of the lowest concentration, where the surface tension was almost constant and just slightly lower than that of water (72.06 ± 0.87 mN/m).

For WE concentrations higher than 38 mg/L, the initial slopes at short adsorption times exhibited a behaviour that could not be considered dependent on the extract content, probably also due to the poor sensitivity of the technique at low scales. This behaviour is opposite to what happens with well recognized surface-active molecules like β-lactoglobulin and β-casein, where the initial slopes vary according to their concentrations in the water phase [56].

For the MPE extracts (Figure 1b), the same behaviour was observed; however, it is interesting to note that at the concentration of 1000 mg/L, a slight increase in the surface tension occurred after 20 min of adsorption, probably due to the formation of aggregates with lower surface properties.

**Figure 2:**
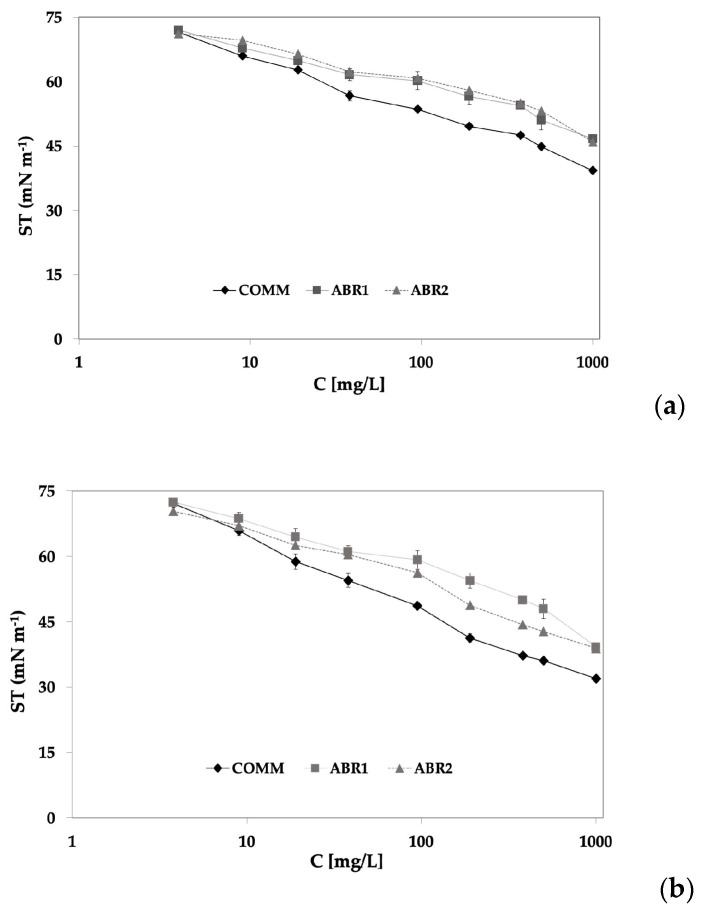
Air–water surface tension of saffron extracts (COMM, ABR1, and ABR2) extracted with H_2_O (**a**) and MeOH/MeCN (**b**) at increasing concentrations.

Both WE and MPE extracts exhibited surface activity with a dose-dependent behaviour, and this applies to all saffron samples under investigation, as could be observed in Figure 2, where the ST values of both extract types obtained at 1 h of adsorption time are reported as a function of the extract concentration. In general, ST showed an initial and more significant decrease when small concentrations (up to 65 mg/L) of the extracts were added, followed by a reduced decrease at higher concentrations (Figure 2a). For concentrations lower than 38 mg/L, no differences were evidenced among the three WE extracts (Figure 2a); however, for higher concentrations, the trends of the ABR1 and ABR2 extracts were comparable and higher than the COMM aqueous extract. The correlation between the surface tension and the crocin contents of the extracts showed a linear trend with an R^2^ value above 0.75, except for the COMM sample that showed an R^2^ around 0.65.

As regards the MPE extracts (Figure 2b), a trend of the ST change as a function of the extract concentration was obtained that was similar to that of corresponding WE, with small differences. In particular, the initial steep decrease of ST occurred with a larger extract concentration (up to 200 mg/L) and above this concentration, the ST decrease was slower, as the air–water interface was approaching saturation and amphiphilic molecules were crowded at the interfacial layer.

The COMM saffron showed the highest ability to decrease the ST also when the MPE extract is taken into account (Figure 2b). However, in this case, some differences were also found between the ABR1 and ABR2 extracts, with the latter showing a higher tendency to locate at the air–water interface than the former.

It should be pointed out that in both the WE and MPE extracts, in the range of concentrations tested, no sharp kink or any marked slope change were observed as usually occurs with surfactants approaching their critical micellar concentration.

Generally, the saffron extracts characterized by medium polarity were more surface active than the corresponding aqueous extracts. The maximum surface tension depression was observed with the MPE COMM extract, with ST values comparable to those that are usually obtained with low molecular weight surfactants such as Tween 20 at concentrations above 2 × 10^−6^ mol/L [56].

The higher surface activity of the MPEs with respect to the WEs can be ascribed to the extracting solvent properties and, in particular, to its polarity, allowing higher extraction of amphiphilic compounds.

**Figure 3:**
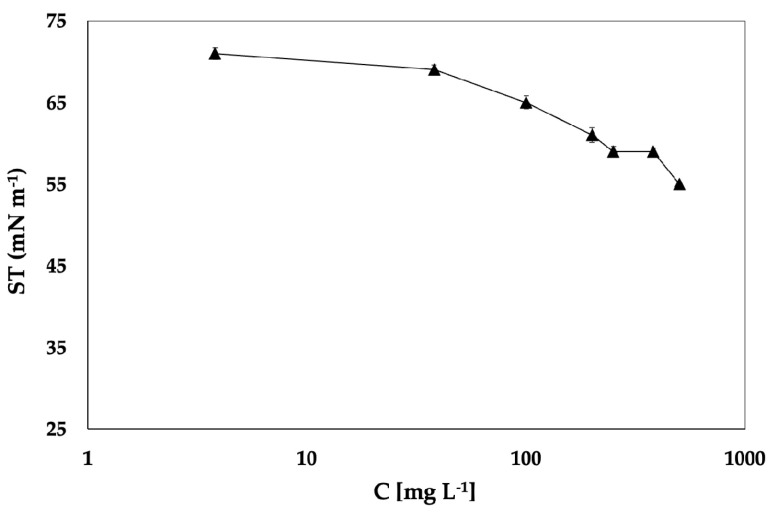
Air–water surface tension of the crocin standard mix at increasing concentrations.

However, the differences in the ST isotherms of the WE and MPE observed in Figure 3 could not be attributed to the crocin contents in the extracts, as no significant differences in their contents and patterns was determined (Table 3).

In the literature, crocin is defined as a bola-amphiphile, i.e., an amphiphilic molecule that has hydrophilic groups at both ends of a sufficiently long hydrophobic hydrocarbon chain, and its moderate surface activity has already been reported; in particular, the surface activity referred only to the trans4 that showed surface and aggregation properties in water [34]. To the authors’ knowledge, no studies on the surface properties of crocins mixtures are available in the literature; thus, both to complement the results and to deepen the understanding of their effects on the surface activity of saffron extracts, measurements of ST of the crocin standard mix solutions at different concentrations were also carried out. The air–water surface tension of the crocin standard mix as a function of the crocin concentration on the logarithmic scale is thus reported in Figure 3. The standard mix showed a concentration-dependent behaviour, and the lower ST value (53.00 mN/m ± 0.77) was reached for the aqueous solutions at 500 mg/L. The *a*/*w* interface reached a pseudo-saturation level at a concentration of about 250 mg/L, and above this concentration, a plateau state seems to be reached with a very limited further decrease in ST up to 500 mg/L. The reported minimum ST value is in agreement with data reported in the literature [34].

These results highlighted that at the same concentrations, the crocin standard showed a higher value of ST than saffron extracts. Furthermore, the COMM sample, which is characterized by a lower total crocin content than the other samples under investigation, showed a significantly higher surface activity for both WE and MPE. It can thus be hypothesized that the crocin content is not the only factor that affects saffron extracts’ surface properties, and the contributions of other surface-active components, like proteins or flavonoids, cannot be excluded [57,58]. The presence of other amphiphilic compounds in the saffron extracts could influence the adsorption behaviour and determine competitive or synergistic phenomena during the mass transfer at the interface, with a significant effect on the surface activity of the extracts.

These aspects need further investigations, such as the isolation/separation of compounds based on their polarity and water/lipid affinity and the investigation of their corresponding model systems.

### 2.4. A/W Surface Tension of Mixed Systems

The analysis of the kinetics of surfactant adsorption becomes more complex when different surface-active compounds are present in the solution; surfactant molecules can take part in molecular reorientation at the interface, undergo a surface transformation to another conformation, or form aggregates or clusters either in the bulk phase or in the interfacial adsorbed layer [59]. Moreover, in a dynamic situation, competitive adsorption and/or can take place between the surfactant and other surface-active molecules present in the system [42].

Surface tension measurements were carried out on mixed systems made of saffron WEs or MPEs and a low-molecular-weight surfactant, Tween 20, at different concentrations. This compound was chosen as a model surfactant with well-known properties and colloidal behaviour [59,60].

Based on the Tween 20 surface tension isotherm and considering its critical micellar concentration (CMC) of 12.27 mg/L, the following concentrations of Tween 20 were selected, 1.23 × 10^−4^, 4.91 mg/L, and 123 mg/L, to investigate the competitive adsorption in presence of saffron extracts at different saturation conditions of Tween 20 at the air–water layer. Then, for each fixed concentration of Tween 20, three different concentrations of saffron WE/MPE were used: 3.8 mg/L, 38 mg/L, and 380 mg/L.

**Figure 4:**
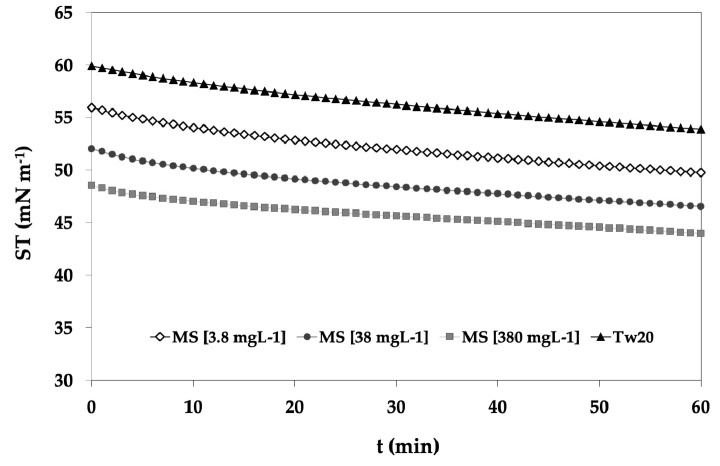
Adsorption kinetics of the mixed system of Tween 20 at [4.91 mg/L] and the WE of the ABR1 sample at increasing concentrations [3.8, 38, and 380 mg/L]. MS = mixed system; Tw20 = Tween 20.

In Figure 4, the ST evolution of Tween 20 (4.91 mg/L) and the corresponding mixed system Tw20-WE (ABR1) at different concentrations as a function of time is reported. The results of the kinetics showed that the addition of the saffron extract to the Tw20 solution resulted in a decrease in the surface tension, and the effect was dependent on the amount of saffron extract added; moreover, the initial slopes at low adsorption times seemed to be affected by the extract concentration, especially at the highest amount, probably due to the formation of aggregates that were slower in reaching the interface. The other mixed systems of the different saffron extracts showed a similar trend, but the surface tension of the MPE of the COMM sample decreased faster than observed for all other solutions.

In Table 4, the surface tension values of the saffron extracts at three increasing concentrations (3.8, 38, and 380 mg/L) and the mixed system (MS) (Tw20–WE or MPE) are reported. At the lowest Tw20 concentration (Table 4), the addition of both the WE (a) and MPE (b) caused a sensitive decrease in the surface tension, especially at the highest extract concentration (380 mg/L), with a dose-dependent behaviour. As for the extracts by themselves, also in the presence of Tw20, the MPE was more effective at decreasing the surface tension with respect to the WE; the same comment can be made for COMM samples compared to ABR1 and ABR2, confirming the surface tension results for the individual extracts. No significant differences were observed between the saffron extracts and the mixed system at the lowest concentration of surfactant.

When the Tw20 concentration was increased to a value of 4.91 mg/L that was still lower than the CMC, the addition of both the WE and MPE resulted in a slight yet significant decrease in the surface tension, with a dose-dependent behaviour also in this case (Table 4). Co-adsorption of Tw20 and saffron extract could have occurred, considering the lower surface tension of the mixed system compared to the pure Tw20 solution. In the saturated system (Tw20 = 123 mg/L), no significant effects were observed on all the systems under investigation. The behaviour observed can be related to the different saturation degrees of the interfacial layer: when the Tw20 concentration was low, a very small coverage of the *a*/*w* interface by Tw20 molecules occurred, allowing the adsorption of the extract compounds with tensioactive properties. Food extracts contain different surface-active ingredients like proteins, phenolic compounds, and other molecules that could interact and lead to the formation of complexes with higher or lower surface properties. In the literature, several works have been reported on the co-adsorption of Tween 20 and proteins, such as bovine serum albumin, β-casein and β-lactoglobulin, and the formation of complexes that have shown different behaviours at *a*/*w* and *o*/*w* interfaces with respect to the single components [61,62,63].

Surface tension measurements were also carried out on mixed systems made of the crocin standard mix and Tween 20 surfactant to investigate any competitive adsorption phenomena at the air–water layer under different saturation conditions of Tw20. The selected concentrations of Tw20 were 1.23 × 10^−4^ and 4.91 mg/L, which are lower than CMC.

**Figure 5:**
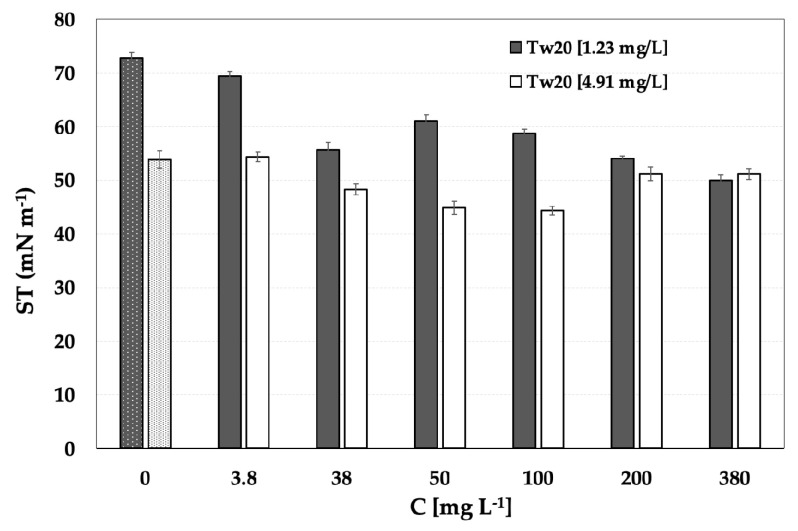
Surface tension of the mixed system containing a fixed amount of Tween 20 [1.23 × 10^−4^; 4.91 mg/L] and increasing concentrations of the crocin standard mix (3.8–380 mg·L^−1^). The bars to the left of the figure represent the control system containing only Tween 20.

In Figure 5, the surface tension evolution of the mixed system of Tween 20–crocin standard mix as a function of the crocin concentration is reported. The trend at the lower concentration of surfactant is similar to that obtained with the sole standard, considering the very low concentration of surfactant.

At a higher concentration of surfactant, the system was more effective at decreasing the surface tension with respect to the lower Tw20 concentration; this could be due to the co-adsorption of crocins and Tw20 molecules at the interface. The *a*/*w* interface reached a pseudo-saturation level at a concentration of about 50 mg/L, and above this concentration, a plateau state seemed to be reached with a very limited increase in the ST at the higher concentrations of the standard, probably due to the formation of some crocin–Tw20 complexes that are less surface active. 

The ST values are higher than the Tw20–saffron extract mixed systems and confirmed the presence of other compounds in the extracts with surface-active properties.

## 3. Materials and Methods

### 3.1. Materials

Three different saffron samples were collected: one commercial sample (COMM, Tre Cuochi, Bonetti S.p.a., MI, Italy) bought at a local supermarket and used as control and two Italian samples (ABR1 and ABR2, Abruzzo, Italy) kindly provided by local producers (Peltuinum S.r.l. L’Aquila, Italy). The latter samples were dried using a low drying temperature (~45 °C) and, upon arrival, the stigmas underwent manual grinding in a dry box up to a fine and homogeneous powder. The commercial sample was already sold as a powder, and detailed information on origin and drying process was not available. After the ISO quality analysis (according to ISO 3632-1:2011), all samples were freeze dried to remove any residual water and stored at −18 °C until analysis.

Methanol (MeOH), acetonitrile (MeCN), Tween 20 (CAS No. 0009005645) and the crocin standard mix (CAS No. 42553-65-1) were purchased at analytical grade from Sigma Aldrich (St. Louis, MO, USA).

### 3.2. Saffron Extract Preparation

Liquid–solid extractions were carried out using two different solvents, in particular, MilliQ H_2_O (WE, polar extract) and a solvent mixture made of MeOH/MeCN (50:50, *v*/*v*) (MPE, medium polarity extract).

#### 3.2.1. H_2_O Extract

A total of 50 mg of saffron sample was dispersed in 100 mL of MilliQ water and subjected to magnetic agitation for 2 h at room temperature under dark conditions. Thereafter, the extract was centrifuged at 4000 rpm for 10 min and filtered through filter paper. Finally, the extract was dried under vacuum (Laborota 4000 eco-Heidolph, Schwabach, Germany) at 40 °C until a constant weight was reached. Nitrogen gas was flushed into the flasks, which were then stored at −32 °C until use.

#### 3.2.2. MeOH/MeCN Extract

The medium polarity extract was obtained from a dispersion of 100 mg of saffron in 20 mL of MeOH/MeCN (50:50, *v*/*v*) subjected to an ultrasonication in a water bath at frequency 50 KHz (Labsonic LBS1, Falc instruments, Treviglio, BG, Italy) for 22 min at 20 °C. The sample was centrifuged at 4000 rpm for 10 min, filtered through 0.45 µm nylon syringe filter with a diameter of 25 mm (Waters, Milford, MA, USA), and then dried under vacuum (Laborota 4000 eco-Heidolph, Schwabach, Germany) at 40 °C until a constant weight was reached. Nitrogen gas was flushed into the flasks, which were then stored at −32 °C until use.

### 3.3. Quality Evaluation by ISO 3632-1

Saffron samples were analysed according to the ISO 3632-1:2011 [53] procedure, with some modifications, as reported by Rocchi et al. (2018) [23]. Briefly, 10 mg of saffron sample was dispersed in 2 mL of distilled water and subjected to Ultrasound-Assisted Extraction (USAE) for 15 min at T = 25 °C. The extracts were centrifuged and analysed by a spectrophotometer (Perkin-Elmer Lambda Bio 20 UV–Visible, Waltham, Massachusetts, US). The absorbance at 257, 330 and 440 nm of the saffron extract, diluted at 1% *w*/*w* in water, was evaluated in a 1 cm path length quartz cell; distilled water was used as reference. 

Then, an aliquot of 15 mg of each sample was exactly weighted and put in an oven at 103 °C for 16 h. The moisture and volatile content is expressed as a percentage of the initial sample and was computed by the following Equation (1):

W_MV_ = (initial weight − final weight)/(initial weight) × 100 (1)

ISO quality indexes were then computed according to the following Equation (2):

A^1^_1cm_(λ_max_) = (D − 10000)/(m × (100 − W_MV_)) (2)

where D is the specific absorbance at the fixed wavelengths; m is the mass of the saffron sample (in g); W_MV_ is the moisture and volatile content of the sample, expressed as a mass fraction.

Analyses were carried out in triplicate on different aliquots of each saffron sample.

### 3.4. Analysis of Crocins

#### 3.4.1. Spectrophotometric Analysis

Saffron extracts at a fixed concentration of 10 mg/L were submitted to a UV-Vis spectrophotometric analysis in a 1 cm path length quartz cell using a spectrophotometer (Jenway 6305 UV/VIS, Staffordshire, UK) at the three fixed wavelengths, 257, 330, and 440 nm, corresponding to the λmax of picrocrocin, safranal, and crocins, respectively.

#### 3.4.2. High-Performance Liquid Chromatographic (HPLC) Analysis

HPLC equipment composed of an Agilent Technologies 1200 series instrument (Agilent, Milano, Italy), with a G1322 degasser, a G1311A quaternary pump, a G136A column thermostat, and autosampler injection system connected to a diode array type of UV/VIS detector (DAD, Agilent Technologies 1200 series, Agilent Technologies, Milano, Italy) was used for the analysis. The system was controlled with Agilent ChemStation for Windows (Agilent Technologies, Milano, Italy).

The target analytes were separated using a reverse-phase Kinetex C18 Phenomenex column (Torrance, CA, USA) (2.6 μm C18, 100 × 3 mm).

**Figure 6:**
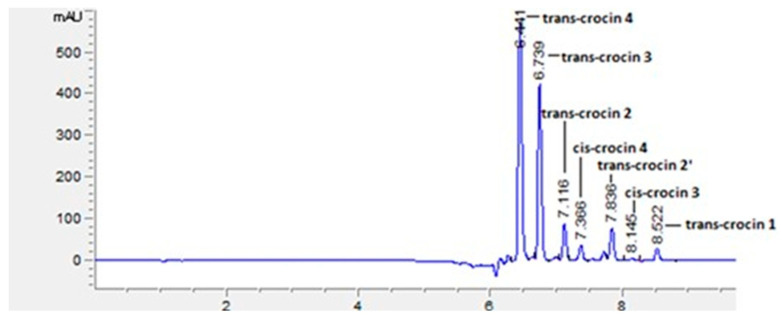
Example of a chromatogram of crocin isomers detected by HPLC-DAD.

The mobile phases were water (Phase A) and acetonitrile (Phase B); the flow rate was 0.4 mL/min and analyte separation was performed using gradient elution: for 0.1 min, phase B was maintained at 5%, then increased to 50% in 5 min and kept at 100% for 5 min; then increased again to 95% in 1 min; and the system was brought back to the initial conditions in 1 min and kept for 4 min. Analyte separation occurred in 8.5 min. All the compounds were detected at 440 nm. An example of the resulting chromatogram with all detected crocins is reported in Figure 6.

### 3.5. Surface Tension Measurements

#### 3.5.1. Solution Preparation

Air–water (*a*/*w*) surface tension was measured between air and the saffron extracts (WE and MPE) at concentrations in the 3.8–1000 mg/L range, which were prepared by diluting the 1000 mg/L stock solution with MilliQ water.

Preliminary measurements of aqueous phase *a*/*w* interface without the addition of saffron extracts were performed and used as control. The Tween 20 surface tension isotherm was also determined by measuring the *a*/*w* surface tension of solutions at different concentrations prepared in MilliQ water from a stock solution (10,000 mg/L).

For the mixed binary extract/surfactant systems, saffron extracts were mixed with Tween 20 (Tw20) in different weight ratio (Table 5).

Based on the Tw20 surface tension isotherm, the selected concentrations of the Tw20 surfactant were [1.23 × 10^−4^ mg/L]; [4.91 mg/L] and [123 mg/L]; at each concentration of Tw20, three different concentrations of saffron extracts were added ([3.8 mg/L]; [38 mg/L]; and [380 mg/L]). The pure Tw20 systems at the selected concentrations were measured and taken as controls for the mixed systems containing the saffron extracts.

#### 3.5.2. Air–Water Measurements

Air–water surface tension (ST) measurements were performed at 20 °C using an Attension Sigma 700/701 tensiometer (Biolin Scientific Oy, Tietäjäntie, Espoo, Finland) equipped with a Wilhelmy plate (width: 19.62 mm-thickness: 0.1 mm). Measurements were carried out on Tween 20 solutions, WE and MPE, and saffron extract–Tween 20 mixed systems at different concentrations and weight ratios.

After calibration (calibration weight 1793 mg) and the measurement of surface tension of water (≃72.8 mN/m, reference), a volume of 25 mL was put in the instrument vessel (d: 46 mm) positioned in the thermostatic vessel holder. Surface tension (ST) data were collected from the time the Platinum Wilhelmy Plate got in contact with the liquid surface of the solution and every 60 s thereafter until 60 min, a time span during which the air–water surface tension of the liquid reached a pseudo-equilibrium condition (i.e., the variation of ST was lower than 0.001 every min). Adsorption kinetics by the change in ST as a function of time were determined for each extract along with the ST value. Data that are reported are those collected at the equilibrium point, after 60 min of adsorption at the air–water interface.

### 3.6. Statistical Analysis

All experiments were carried out in triplicate; the results are reported as means and standard deviations. A one-way analysis of variance (ANOVA) and Tukey’s test were used to establish the significance of differences among the mean values at the 0.05 significance level; the analyses were carried out by XLSTAT software, version 2022.1 (Addinsoft SARL, New York, NY, USA).

## 4. Conclusions

Two different solvents (H_2_O and MeOH/MeCN) with different polarities can cause the selective extraction of compounds that can differ in their chemical properties, including their polarity and this could also have an effect on the extraction of the more amphiphilic ones; therefore, different behaviours of the molecules at the air–water interface were observed. In this work, the attention was focused on crocins, hydrophilic carotenoids present in saffron that are one of the parameters that contribute to the quality of the spice and are responsible for the characteristic orange–yellow colour. The kinetics and degree of crocin adsorption can be relevant for many important applications in various fields, such as in pharmaceutics and in food formulation and processing, such as emulsification.

Results showed that the crocin content is not the only factor responsible for the surface-active properties of the extracts, and the presence of other surface-active components, like proteins or flavonoids, can be hypothesized considering that the two extracts (WE and MPE) showed the same crocin content.

Both saffron extracts (WE and MPE) decreased the surface tension at the air–water (*a*/*w*) interface with a dose-dependent behaviour. In particular, the MPE presented a higher surface activity than the WE. In the mixed Tween 20–saffron extract systems, the surface properties behaved differently on the basis of the amount of Tween 20 present in the mixture. At Tween 20 concentrations lower than the CMC, the addition of the saffron extracts significantly improved the surface properties of the systems; however, no significant effect was detected when the amount of Tween 20 was over the CMC.

These results contribute to a better understanding of saffron extracts’ behaviours in emulsified systems, since crocins, water-soluble carotenoids, facilitate their use in food. Further studies are needed to evaluate the technological functionalities of saffron extracts in presence of other emulsifiers (i.e., whey proteins and polysaccharides), as well as to deepen the understanding of the compositional factors that contribute to their surface properties.

## Figures and Tables

**Table 1:** Quality parameters and categories of saffron samples under investigation according to ISO 3632-1: picrocrocin λmax = 257 nm; safranal λmax = 330 nm; and crocins λmax = 440 nm. ^a–c^ Results followed by a letter of the same case were not significantly different according to Tukey’s HSD post hoc test (*p* > 0.05).

	$E_{1\mathrm{cm}}^{1\%}$			
	Flavour Strength	Aroma Strength	Colouring Strength	ISO Category
	257 nm	330 nm	440 nm	
COMM	112 ^b^	55 ^a^	276 ^b^	I
ABR1	118 ^a^	38 ^b^	288 ^a^	I
ABR2	100 ^c^	25 ^c^	263 ^c^	I

**Table 2:** UV-Vis absorbance values of WE and MPE saffron extracts at selected wavelengths: 257 nm, 330 nm, and 440 nm. Results followed by letters of the same case were not significantly different according to Tukey’s HSD post hoc test (*p* > 0.05). ^a,b^ Lower case letters are for the same type of extract and same wavelength and ^A,B^ upper case letters are for the two extracts and same wavelength.

	WE			MPE		
	Picrocrocin	Safranal	Crocins	Picrocrocin	Safranal	Crocins
	257 nm	330 nm	440 nm	257 nm	330 nm	440 nm
COMM	0.104 ± 0.016 ^aA^	0.061 ± 0.005 ^aA^	0.199 ± 0.038 ^bB^	0.101 ± 0.037 ^aA^	0.054 ± 0.005 ^aA^	0.248 ± 0.016 ^bAB^
ABR1	0.102 ± 0.012 ^aA^	0.043 ± 0.002 ^bAB^	0.249 ± 0.017 ^aAB^	0.116 ± 0.007 ^aA^	0.051 ± 0.003 ^aA^	0.250 ± 0.014 ^bAB^
ABR2	0.113 ± 0.013 ^aA^	0.037 ± 0.016 ^bAB^	0.234 ± 0.037 ^aAB^	0.134 ± 0.008 ^aA^	0.031 ± 0.001 ^bB^	0.315 ± 0.007 ^aA^

**Table 3:** The quantification of the trans4, the % relative area (computed on the total area of the crocins) of the seven crocin isomers in the saffron samples under investigation and the total chromatographic crocin area. The coefficient of variation (CV) is under 9% for all samples. ^a–d^ Results followed by letters of the same case were not significantly different according to Tukey’s HSD post hoc test (*p* > 0.05).

			Relative Area (%)							Total Area *
		trans4 (mg/L)	trans4	ci4	trans3	cis3	trans2	trans2 II	trans1	
WE	COMM	37.05 ± 9.25 ^d^	37.77	4.40	32.55	10.29	7.11	3.56	4.28	1329.55 ^b^
	ABR1	130.47 ± 17.22 ^a^	58.88	5.17	24.83	6.40	1.38	2.20	1.11	2957.50 ^a^
	ABR2	92.19 ± 6.89 ^c^	48.44	3.41	30.90	7.73	5.12	2.23	2.14	2546.35 ^a^
MPE	COMM	107.00 ± 12.65 ^bc^	54.11	5.26	23.82	9.50	2.15	2.94	2.19	2483.01 ^a^
	ABR1	117.92 ± 12.08 ^ab^	57.38	4.66	25.78	6.88	2.11	2.43	0.72	2744.35 ^a^
	ABR2	122.66 ± 6.87 ^abc^	56.05	6.50	21.69	9.20	1.65	2.92	1.94	2921.90 ^a^

* mAU.

**Table 4:** Surface tension of Tw20 [1.23 × 10^−4^; 4.91 and 123 mg/L] (control), saffron extracts at three increasing concentrations (3.8, 38, and 380 mg/L) and the mixed system (MS) (Tw20–WE or MPE). Results followed by letters of the same case were not significantly different according to Tukey’s HSD post hoc test (*p* > 0.05). Letters from a–c are for the analysis in column for each system; letters from A–C are for the analysis in rows for each saffron extract concentration.

		Saffron Extract Concentration	Surface Tension (mN m^−1^)			
			Extract	MS Tw20 (1.23 × 10^−4^ mg/L)	MS Tw20 (4.91 mg/L)	MS Tw20 (123 mg/L)
		[0 mg/L]	-	72.60 ± 0.90 ^aA^	51.97 ± 0.76 ^aB^	37.65 ± 0.20 ^aC^
WE	COMM	[3.8 mg/L]	71.56 ± 0.92 ^aA^	72.25 ± 0.33 ^aA^	49.46 ± 0.01 ^abB^	36.54 ± 0.25 ^aC^
		[38 mg/L]	62.73 ± 0.36 ^bA^	63.28 ± 1.29 ^bA^	46.73 ± 0.68 ^bB^	36.52 ± 0.44 ^aC^
		[380 mg/L]	47.52 ± 0.88 ^cA^	49.45 ± 0.19 ^cA^	40.71 ± 1.02 ^cB^	35.86 ± 0.01 ^aC^
		[0 mg/L]	-	72.60 ± 0.90 ^aA^	51.97 ± 0.76 ^aB^	37.65 ± 0.20 ^aC^
	ABR1	[3.8 mg/L]	72.06 ± 0.87 ^aA^	72.24 ± 0.48 ^aA^	49.73 ± 0.59 ^bB^	35.99 ± 0.05 ^aC^
		[38 mg/L]	64.65 ± 0.49 ^bA^	65.68 ± 1.12 ^bA^	46.51 ± 0.25 ^cB^	36.16 ± 0.14 ^aC^
		[380 mg/L]	54.43 ± 0.65 ^cA^	53.46 ± 0.60 ^cA^	43.97 ± 0.51 ^dB^	36.21 ± 0.20 ^aC^
		[0 mg/L]	-	72.60 ± 0.90 ^aA^	51.97 ± 0.76 ^aB^	37.65 ± 0.20 ^aC^
	ABR2	[3.8 mg/L]	71.23 ± 1.42 ^aA^	72.24 ± 0.10 ^aA^	50.49 ± 0.82 ^abB^	36.65 ± 0.32 ^aC^
		[38 mg/L]	66.34 ± 0.21 ^bA^	66.24 ± 0.65 ^bA^	47.36 ± 0.86 ^bB^	36.26 ± 0.22 ^aC^
		[380 mg/L]	54.92 ± 1.28 ^cA^	54.85 ± 0.15 ^cA^	46.81 ± 0.71 ^bB^	35.88 ± 0.21 ^aC^
		[0 mg/L]	-	72.60 ± 0.90 ^aA^	51.97 ± 0.76 ^aB^	37.65 ± 0.20 ^aC^
MPE	COMM	[3.8 mg/L]	72.12 ± 0.73 ^aA^	71.66 ± 0.92 ^aA^	48.08 ± 0.26 ^bB^	36.83 ± 0.14 ^aC^
		[38 mg/L]	58.52 ± 1.63 ^bA^	58.17 ± 3.93 ^bA^	44.50 ± 0.53 ^cB^	36.15 ± 0.17 ^aC^
		[380 mg/L]	37.20 ± 0.19 ^cA^	40.18 ± 2.80 ^cA^	38.16 ± 0.50 ^dA^	34.96 ± 0.42 ^aA^
		[0 mg/L]	-	72.60 ± 0.90 ^aA^	51.97 ± 0.76 ^aB^	37.65 ± 0.20 ^aC^
	ABR1	[3.8 mg/L]	72.42 ± 0.64 ^aA^	68.05 ± 0.72 ^abB^	48.86 ± 0.99 ^abC^	36.89 ± 0.21 ^aD^
		[38 mg/L]	64.10 ± 0.06 ^bA^	63.21 ± 0.02 ^bA^	47.67 ± 0.06 ^abB^	36.84 ± 0.19 ^aC^
		[380 mg/L]	50.01 ± 2.59 ^cA^	47.93 ± 0.19 ^cA^	43.36 ± 0.71 ^bAB^	36.95 ± 0.37 ^aB^
		[0 mg/L]	-	72.60 ± 0.90 ^aA^	51.97 ± 0.76 ^aB^	37.65 ± 0.20 ^aC^
	ABR2	[3.8 mg/L]	70.38 ± 1.38 ^aA^	70.06 ± 0.33 ^aA^	48.03 ± 0.11 ^abB^	36.73 ± 0.12 ^aC^
		[38 mg/L]	62.41 ± 0.12 ^bA^	61.53 ± 0.13 ^bA^	47.67 ± 0.06 ^bB^	36.41 ± 0.02 ^aC^
		[380 mg/L]	44.35 ± 1.35 ^cA^	45.21 ± 0.99 ^cA^	44.48 ± 1.09 ^cA^	36.68 ± 0.22 ^aB^

**Table 5:** Different concentrations and weight ratios of different saffron extract–Tween 20 mixed systems.

	Tw20 (1.23 × 10^−4^ mg/L)	Tw20 (4.91 mg/L)	Tw20 (123 mg/L)
Saffron Extract	Tw20–Saffron Extract		
[3.8 mg/L]	1:31,000	1:0.77	1:0.03
[38 mg/L]	1:310,000	1:7.7	1:0.3
[380 mg/L]	1:3,100,000	1:77	1:3

## Data Availability

Data available on request.
